# Simplifying the Process of Going From Cells to Tissues Using Statistical Mechanics

**DOI:** 10.3389/fphys.2022.837027

**Published:** 2022-03-25

**Authors:** Jagir R. Hussan, Mark L. Trew, Peter J. Hunter

**Affiliations:** Auckland Bioengineering Institute, University of Auckland, Auckland, New Zealand

**Keywords:** systems biology, multiscale modeling, statistical mechanics, functional tissue unit, physically consistent modeling

## Abstract

The value of digital twins for prototyping controllers or interventions in a sandbox environment are well-established in engineering and physics. However, this is challenging for biophysics trying to seamlessly compose models of multiple spatial and temporal scale behavior into the digital twin. Two challenges stand out as constraining progress: (i) ensuring physical consistency of conservation laws across composite models and (ii) drawing useful and timely clinical and scientific information from conceptually and computationally complex models. Challenge (i) can be robustly addressed with bondgraphs. However, challenge (ii) is exacerbated using this approach. The complexity question can be looked at from multiple angles. First from the perspective of discretizations that reflect underlying biophysics (functional tissue units) and secondly by exploring maximum entropy as the principle guiding multicellular biophysics. Statistical mechanics, long applied to understanding emergent phenomena from atomic physics, coupled with the observation that cellular architecture in tissue is orchestrated by biophysical constraints on metabolism and communication, shows conceptual promise. This architecture along with cell specific properties can be used to define tissue specific network motifs associated with energetic contributions. Complexity can be addressed based on energy considerations and finding mean measures of dependent variables. A probability distribution of the tissue's network motif can be approximated with exponential random graph models. A prototype problem shows how these approaches could be implemented in practice and the type of information that could be extracted.

## 1. Introduction

**Figure 1:**
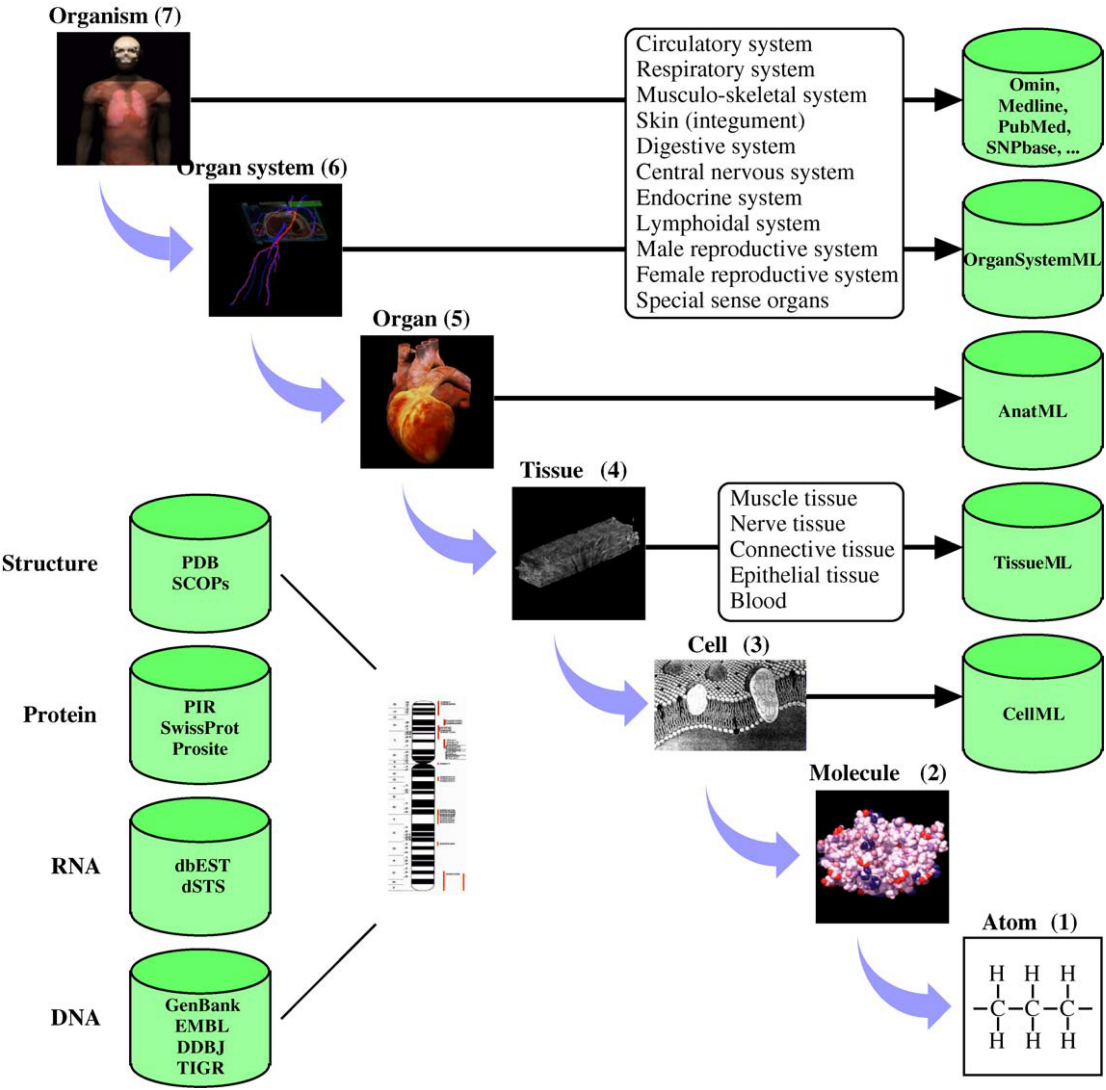
Physiological systems, processes, and corresponding spatial scales encompassed by the Human Physiome Project. The databases hold physiologically relevant data and model information encoded in markup languages such as CellML (see www.cellml.org) and FieldML. The markup languages ensure that models are encoded in a consistent form and allows simulation packages to import the models in a standard format. Reproduced with permission from Hunter (2004).

Digital twins are increasingly becoming critical components of modern life (Tao and Qi, 2019). Much of modern engineering design, analysis and development rely strongly on validated, high fidelity computer models (Wynn and Clarkson, 2018; Lim et al., 2020). These models are not only cost-effective design tools but are critical to understanding long term behaviors. Mirroring these developments in general engineering, there has been significant progress in developing quantitative models of human and animal physiology (Gillette et al., 2021). However, general engineering approaches differ from their biophysical science counterparts in at least one important aspect. Scientific inquiry into complex biophysical functions typically uses reductive methods to tease apart complex mechanisms. Engineering often uses compositional approaches to build a feature rich system (c.f. Ai et al., 2018). This principal lies at the heart of systems biology, where the systems approaches developed in engineering are used to piece together the reductive models of physiological function (Tavassoly et al., 2018). For quantitative understanding of biological systems, digital models often incorporate physical properties at multiple spatial and temporal scales. To achieve this requires the provenance of models and data sources and an ability to seamlessly integrate biophysics at different scales (Hunter and Borg, 2003). To systematically handle system complexity, many model development, data storage and simulation standards have been developed (Hunter, 2020). Figure 1 shows an example of applying systems-based approaches to human physiology from the IUPS Human Physiome project (Hunter, 2004).

Models conforming to these standards should enable algorithmic, automated composition and analysis of physiological systems (Hunter, 2020). In practice there remain challenges to developing useful digital twins. Here we discuss two critical bottlenecks that have stifled progress and propose approaches for addressing them. The bottlenecks are: 1) taming physically inconsistent “language” across composite model scales, especially for conservation of mass and energy; and 2) taming the inevitable growing conceptual and computational complexity to usefully inform scientific inquiry, clinical decision making and support biotechnology development.

## 2. Taming the Tower of Babel

Clinically useful physiological models must do more than just characterize complex anatomical and functional domains. Model parameters need to be linked with *multiscale modeling* to molecular processes where drugs operate. However, many models are developed in response to specific needs such as bridging measurement scale gaps, interpolating or extrapolating missing data or as empirical relationships extracted from big data (for example, using machine learning approaches). Often these models are mutually incompatible. To support inter-model coupling, standards and provenance information have been proposed, ensuring semantically consistent data exchange from model to model (Hunter, 2020). Organ scale models can be built as a hierarchical compositions of finer scale models if spatio-temporal scales can be correctly characterized. The compositional language matters, and anatomical components and physiological systems must satisfy physical principles such as mass-, charge-, and energy balances along with associated thermodynamics. A consistent physical language simplifies the development of algorithmic approaches to compose, analyse and verify composite models.

### 2.1. Physically Consistent Model Development

Bond graphs are a model development framework that is biophysically and thermodynamically consistent (Oster et al., 1971; Gawthrop et al., 2015). The kinetics of most biophysical phenomena can be described using a potential, *u*, as a function of a physical quantity, *q*. The flow, *v* = *dq*/*dt*, and potential, *u*, must satisfy conservation laws. For example, mass or charge conservation for *v* and force balance or Gibbs free energy for *u*. The lumped-matter discipline of electrical engineering (Agarwal and Lang, 2005) is used to define the constitutive relation between the potential *u* and physical quantity *q*. Since power is independent of the physical domain (Broenink, 1999), the formalism enables seamless and consistent integration of multiple physical domains through notional use of concepts such as junctions, transformers and gyrators. A bond graph description of a biophysical process produces models that have both physical and biophysical interpretations (Gawthrop et al., 2015). Models can be correctly and algorithmically coupled, and the composite model is itself a physically consistent bond graph, biologically interpretable and causally transparent (Cobos Mendez et al., 2020; Shahidi et al., 2021). Supplementary Figure 3 shows an example bond graph model of a biophysical cardiac cell action potential.

## 3. Taming Towering Complexity

Bond graphs are a consistent framework for addressing multiple parameter descriptions and ensuring consistent units and conservation laws. But this does not mitigate the need to specify appropriate constitutive relations. Some of these relationships can be determined from experimental data; however, often a multiscale model approach is used to determine constitutive parameters by simulating subscale models. Additionally, bond graphs are zero-dimensional so space-time discretizations are required, with each discrete unit encapsulated in a bond graph and each of these instances coupled. This rapidly becomes a problem of towering complexity. In the following discussion, multiscale modeling of cardiac electrophysiology is used as an exemplar for addressing this challenge and to illustrate an approach for unlocking emergent biophysical insights.

### 3.1. Functionally Dependent Cellular Interconnections

The adult human heart contains several billion myocytes. Computationally tractable mathematical models based on homogenization techniques (Tung, 1978) are currently the gold standard for simulating electrochemical conduction in cardiac tissue (Franzone et al., 2014). For solving, the cardiac domain is spatially discretized into blocks representing the activity of groups of myocytes. For example, if a domain is discretized into a regular lattice of blocks with edge length 0.25 mm, a block will contain about 1,000 myocytes. State-of-the-art human heart bioelectric models on this scale solve for around 11 billion electrical potentials at each step in time (Potse et al., 2020). The spatially discrete models are coupled to models of myocyte membrane ion transport (typically one homogenized model per block of myocytes) to account for dynamic electrical loading type behavior with cardiac activity. Reaction-diffusion bidomain models assume interwoven extracellular, membrane and intracellular spaces everywhere in the tissue. This restricts the range of problems that can be realistically simulated by the model. For example, simulating the role of spatially localized effects like altered ion channel expression, fibrosis, tissue scarring and so on, in rewiring interconnection topology and the consequent impacts on macroscopic conduction is not possible (Pastore et al., 1999; Qu et al., 2000; Amoundas et al., 2001; Kawara et al., 2001; de Bakker et al., 2005).

### 3.2. Computational Complexity

Solving for dependent variables at many millions of discrete points over an acceptable time frame is a significant issue. Existing models with this capability require specialized software and hardware (Richards et al., 2013; Potse et al., 2020). While it is argued that computational capacity, including specialized processing based around graphical processing units (Kaboudian et al., 2019), will grow, become cheaper and more accessible, data transfer bottlenecks remain and may be limiting factors (Mo, 2018). The drive to capture more features in models keeps pace with developing computational resources and the capacity to clinical or intervention diagnostics by simulating physiological functions in or near real-time has not been realized (Islam et al., 2016; Yip et al., 2018).

### 3.3. Structural Complexity

Coupling topology describes how individual discretized units are inter-connected to reproduce tissue space-time behavior. Cells embedded in a connective tissue matrix sense signals (inter-cellular and systemic) and process the complex information to make decisions enhancing survival (as well as the function) of the whole tissue (Karemaker, 2015; Silvani et al., 2016). For example, these processes rewire excitable cellular pathways, alter refractory periods and so on. Consequently, the process of discretizing tissue and coupling together the dynamics of discrete units must be informed by how the cells within the tissue organize under different conditions. Some of this may not be known a priori. Coupling methods like homogenization (Neu and Krassowska, 1993) model local short-range interactions and not non-local long-range interactions unless they are explicitly handled. Incorrect short and long-range coupling is observed in a disconnect between model and physiology in simulations of tissue level phenomena such as cardiac electrical arrhythmia. The same may also be true if the discretization is too coarse to capture important phenomena. Addressing these challenges requires appropriate interpretation of tissue structure and a digital representation that integrates tissue, cellular, and molecular understanding across multiple scales (Hunter, 2004).

#### 3.3.1. Functional Tissue Unit

Cells embedded in a connective tissue matrix are biophysically required to be within diffusion distance of a capillary blood vessel for access to nutrients, such as oxygen and glucose, and elimination of waste, such as carbon dioxide and urea. Coupled mammalian cells are mostly within 50μ m of a capillary (Renkin and Crone, 1996). Based on this length scale observation, the concept of a functional tissue unit (FTU) was developed to facilitate digital representation of tissue (de Bono et al., 2013). The FTU gives precise dimensions for subdividing tissues based on biophysical constraints, and when it is based on local capillary geometry, it is essentially equivalent to local myofiber orientation (Vignaud et al., 2006). In this context the FTU is aligned with the material axes and this is relevant for both the electrophysiology and mechanics of cardiac tissue. An FTU should capture the kinds of cells that exist within that space and the contributions they make to tissue level phenomena. For example, extracellular molecules such as ions, paracrine signals and so on.

The concept of FTU has been adopted by Human BioMolecular Atlas Program (HuBMAP) project (Snyder et al., 2019). This programme is creating a cellular atlas of the human body. Methods and tools to interconnect cells with organs are being developed (Weber et al., 2020). There are other large scale efforts to map the human body like the ChanZuckerberg Initiative Human Cell Atlas (HCA) (Human Cell Atlas, 2021). The outcomes of these projects will be modular computer models, informed by cellular organization with tissues and quantification of altered organization under disease conditions. There are ongoing attempts to model FTUs as bond graph elements and this is expected to improve spatial discretization and modeling of spatially heterogeneous processes (Hunter, 2020).

Given that FTUs provide a digital representation that integrates tissue, cellular and molecular understanding across multiple scales; a framework to model FTUs and their compositions is required. In the following sections, we show that recent developments in statistical mechanics of networks offer useful insights.

## 4. Statistical Mechanics to the Rescue?

Multiscale models (bondgraph-based or otherwise) are conceptually and computationally demanding. Consequently, it is important to explore other complementary frameworks that are physically measurable and causally transparent. Statistical mechanics is a framework that applies probability theory to large collections of microscopic or atomic particles to explain macroscopic observations. Approaches based on statistical mechanics have been widely and successfully used in physics and engineering to characterize material properties and analyse physical phenomena (Goldstein et al., 1992; Tohei et al., 2006; Scheffer, 2010; Teschendorff and Feinberg, 2021). While atomic elements in physics and engineering are comparatively simple compared to many biological cells of interest, one key principle stands out in the context of multicellular organ models: *maximum entropy*.

It is often assumed that cells or biological systems have identical processes and this assumption ensures that uniform spatial discretizations are valid and is common in tissue models (like the reaction-diffusion bidomain models of cardiac electrophysiology described previously). However, cells embedded in a multicellular environment exhibit intercellular variations due to fluctuations in gene expression, protein synthesis or access to local nutrient concentration (Nelson and Masel, 2017). These fluctuations force cells to explore different metabolic states other than the “optimal” ones. Ideally, cells evolve interactions so that stress generated to access resources from a shared capillary is close to minimal for most cells in the functional tissue unit (Aktipis, 2016). Many intercellular coupling configurations could reduce generated stress and such variability needs to be considered in models.

### 4.1. Statistical Mechanics of Solid Tissues

A functional tissue unit provides guidance for spatial discretizations and digital representation of tissue. As the FTU is defined under biophysical constraints (proximity to blood supply, material orientation, etc.), leveraging the FTU as a basis for spatial discretization confers these geometric features onto the topology of a network, G, characterizing intercellular coupling. Supplementary Section 1 demonstrates the construction of such a network for cardiac ventricular tissue.

As multiple intercellular coupling's could lead to the observed organ-level dynamics, the principle of maximum entropy plays a key role in determining the set of intercellular configurations (G∈s) that best represents the current state of knowledge. Such a set of networks *s* has the largest entropy (Jaynes, 1957), is consistent with known constraints and can accommodate maximum uncertainty with respect everything else (Harte and Newman, 2014). Determining *s* enables modelers to analyse distributions of cells and their function within the tissue and use these coupling configurations to simulate emergent phenomena. Exponential random graphs model (Park and Newman, 2004; Barrat et al., 2008) provide natural and very elegant frameworks to determine *s* that satisfies the principle of maximum entropy.

### 4.2. Exponential Random Graphs Model

Exponential random graph models (ERGMs) express a probability distribution on cell networks that arises from competing forces causing some interactions to be more or less likely, given the state of the rest of the network. Complex dependency patterns can emerge within the network, including large-scale organizations emerging from relatively simple local mechanisms. ERGMs incorporate varying network structure and cell features (biophysical properties or spatial locality) so that a network and cell configuration can be assessed in the context of all other model possibilities (Barrat et al., 2008).

The assumption of ERGMs is there are many possible realizations of a network even if only one is observed empirically. Given a network, G, and features $x_{i}\left(G\right)$ observed on the network, the model defines a probability distribution over them and creates a statistical ensemble - the set of networks G∈s and the distribution P(G).

The probability distribution governing *s* at macroscopic equilibrium is given by (Lusher et al., 2012)


(1) $$P\left(G|\theta ,x\right)=\frac{\exp(\theta^{T}x(G))}{\sum_{G^{\prime }\in s}\exp(\theta^{T}x(G^{\prime }))h\left(G^{\prime }\right)}h\left(G\right).$$

Here G is a particular network (microstate) drawn from the set of potentially observable networks (microstates) $s,G^{\prime }$ is another potentially observable network, **x**↦ℝ^*n*^, is a vector of observations encoding the network properties, θ∈ℝ^*n*^ is a vector of parameters, and h(G) is the reference measure for the distribution.

h(G) characterizes the geometric constraints on the network topology G. Some of these constraints arise from the observed spatial embedding of cells, such as minimum and maximum number of neighbors, and other physiological requirements such as nutrient flow directions. Thus, a network that satisfies these observations will have a high *h* value, where as one that does not satisfy these observations will have low value making it less probable.

The term $\theta^{T}\text{x}\left(G\right)$ is the *ERGM potential*. Identifying it with -βH(G), Equation (1) can be recognized as the Boltzmann distribution of G and the x(G)'s as the energy terms of the Hamiltonian.

Traditionally Markov Chain Monte Carlo methods are used to estimate these parameters, and could be computationally expensive or even intractable for large networks. However, recent developments have reduced parameter estimation time by orders of magnitude and make ERGMs amenable for studying biological systems (Stivala et al., 2020).

In our opinion, these features, such as the ability to handle sparse multi-domain data and operate in an energy-based construct makes ERGMs an attractive framework for studying solid tissues. They link with many powerful tools developed in the statistical mechanics for studying complex systems.

### 4.3. Feasibility Demonstration

To demonstrate the approach, we use a simplified model of electrical arrhythmia as a datasource to construct and analyse ERGMs for various structural configurations. We show that the predicted ERGM potentials characterize the underlying structure and capture node level observations. Details of the models and experimental setup are given in the Supplementary Material.

**Figure 2:**
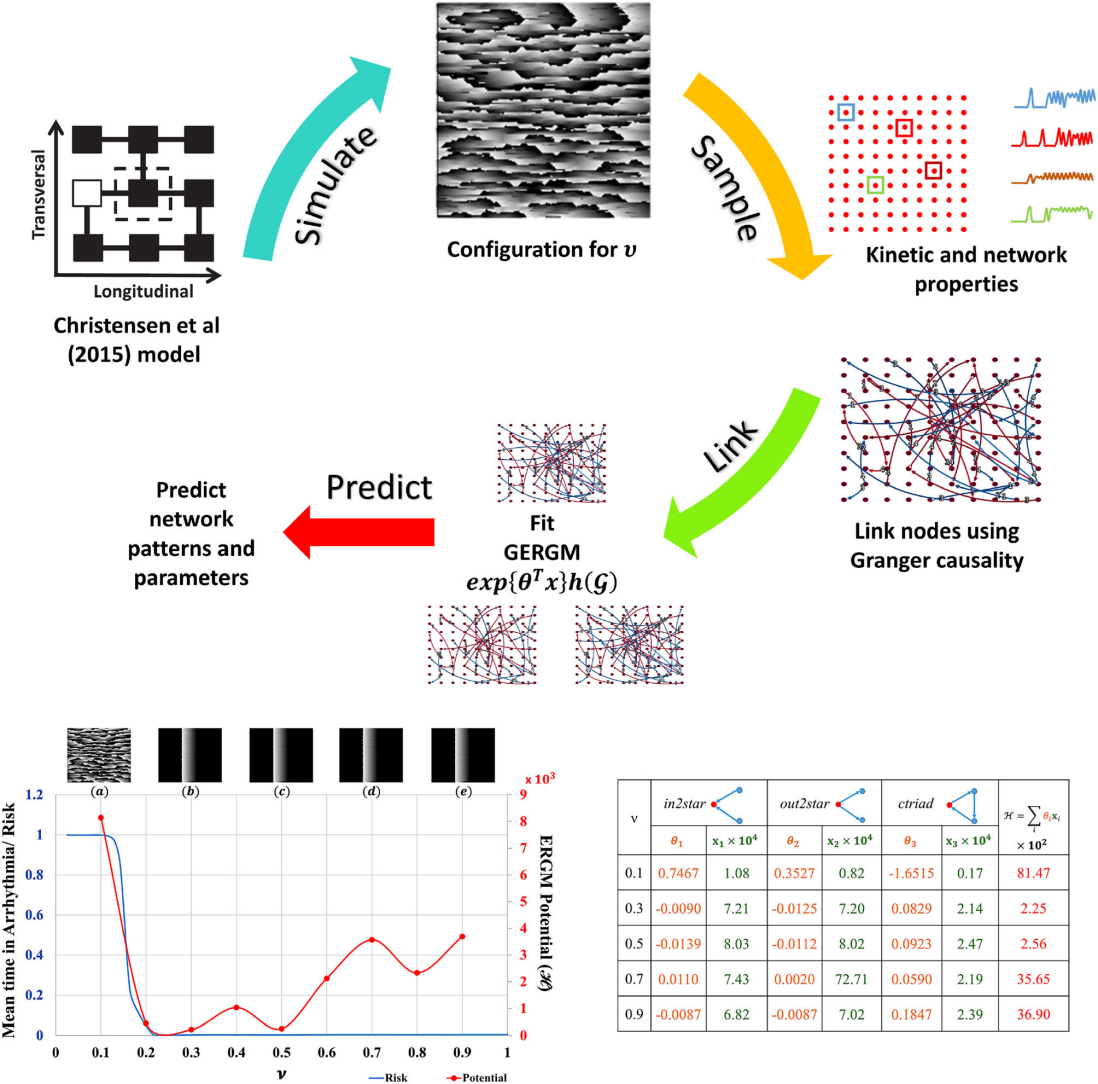
ERGM evaluation. **Top**: Schematic of ERGM generation process. State-transition kinetics for 2D tissue characterized by ν is sampled (red dots), the transition kinetics (a color coded subset along with their locations on the 2D tissue is shown) is used to create the causal network. Other nodal and network observations are also collected. A GERGM model is fit to the causal network to predict networks with similar topological characteristics, nodal and network observations. **Bottom Left**: Model predicted “Mean time in arrhythmia/Risk of arrhythmia” as a function of lateral uncoupling parameter ν. Insets show the plane wave dynamics exhibited by the model at *T* = 1, 000 units for each ν. Each 2D model consists of 200 × 200 cells with a refractory period of 50±5 units, and 20 randomly placed dysfunctional cells that misfire with a probability of 0.05. Pacemaker cells at the left edge self-activate with a period of *T* = 220 units and initiate a planar wave. As ν decreases from 1.0, a transition from planar wave fronts to a system of multiple self-sustaining reentrant circuits (ν ≤ 0.14) is observed. The corresponding ERGM potentials are plotted along with the inset label. **Bottom Right:** GERGM calculated coefficients (θ_*i*_) for the observed network structural characteristics (*x*_*i*_) and the calculated potential. See Supplementary Material for full images and method details.

The Christensen et al. (2015) lattice model of electrical conduction uses parameter ν to alter 2D lateral lattice coupling frequency. At ν = 1 the lattice is fully coupled and at ν = 0 is fully uncoupled (see Figure S5). Electrical potential sequences for a range of ν values from 0.1 to 0.9 were determined. Abstracted network models of these data were built by first subsampling the lattice model connectivity and encoding the lattice cell state values (Figure S5) for each ν onto a 10 × 10 grid of nodes using averages of a 3 × 3 neighborhood. Nodes anywhere in the 10 × 10 grid were functionally linked into a network if their Granger causality [estimated with partial correlations (Runge et al., 2019) of cell state time series] was greater than 0.1 %. The cross-correlation of average cell state time series between connected nodes was the weight for that link. These steps are shown in the top panel of Figure 2. This is the original network G(ν) in Equation 1. The 10 × 10 networks (and others derived from them) are abstractions of the original raw data sets [from the Christensen et al. (2015) models].

Network node motifs were used to encapsulate functional behavior in the abstracted 10 × 10 grid networks. These are the variables used by Generalized ERGM to generate alternative networks from probability distributions that have link weights (functional connectivity) and node weights (structural connectivity in this example) h(G) that correlate with the values in G(ν). Three network motifs relevant to arrhythmic risk were specified: (i) nodes with two incoming network links (*in2stars*), (ii) nodes with two outgoing network links (*out2stars*), and (iii) nodes forming local cyclic networks with two other nodes (*ctriads*). Nodes functioning as hubs with either *Sender* or *Receiver* effects were additional constraints on the GERGM models. GERGM models using these three network motif variables were fit to the original networks G(ν) to find three weight parameters (θ_1_, θ_2_ and θ_3_) given the observed motif counts (*x*_1_, *x*_2_, *x*_3_). Together they can be used to compute an ERGM potential across the equivalent networks at each ν. Existing software tools were used as a black box to solve these problems (Denny, 2016).

Comprehensive methodology for GERGM is beyond the scope of this perspective, but can be found in (Desmarais and Cranmer, 2012). Complete code and parameters used for generating and comparing networks is found in the opensource code base in github.

The results summarised in Figure 2 show that with statistical analysis of networks abstracted from detailed but often inaccessible source data, network potentials (ERGM potentials) can be found to unmask transitions in behaviour (in this case arrhyrthmic risk). Traditional models applied at the relatively coarse scale and resolution of the abstracted networks would not be expected to expose such features.

## 5. Perspective

The richness of human physiology and physiological processes requires a systematic approach to tease out its inner workings. It is important (but challenging) to develop data, modeling, provenance and exchange frameworks that can assimilate multiscale features, respecting physics-based conservation principles while remaining computationally tractable. Physics-based statistical mechanics approaches provide clear and concise principles to investigate complex systems. Recent developments in physics-based machine learning tools have incorporated these principles to explore biophysical mechanisms in empirical data. Exponential random graph models (ERGMs) belong to this category of tools and show promise for improving our understanding and modeling of multiscale physiological processes. ERGMs may play a significant role in developing physiological digital twins.

## Data Availability Statement

The original contributions presented in the study are included in the article/Supplementary Material, further inquiries can be directed to the corresponding author/s.

## Author Contributions

JH and MT wrote the article and performed the simulations. PH developed the concepts around functional tissue units and application of Bondgraphs to physiology. All authors conceived the research project and contributed to the article and approved the submitted version.

## Funding

MT was supported by a grant from the Leducq Foundation. This work was supported by ABI PBRF fund towards publication charges and New Zealand's MBIE for ABI's 12 Labours project.

## Conflict of Interest

The authors declare that the research was conducted in the absence of any commercial or financial relationships that could be construed as a potential conflict of interest.

## Publisher's Note

All claims expressed in this article are solely those of the authors and do not necessarily represent those of their affiliated organizations, or those of the publisher, the editors and the reviewers. Any product that may be evaluated in this article, or claim that may be made by its manufacturer, is not guaranteed or endorsed by the publisher.
