# Supplementary material for: Simplifying the Process of Going From Cells to Tissues Using Statistical Mechanics
Source: Front Physiol. 2022 Mar 25;13:837027. doi: 10.3389/fphys.2022.837027 (PMC8990301; doi:10.3389/fphys.2022.837027)
Supplement: Supplementary file 1 [file Data_Sheet_1.pdf]

Supplementary Material
Simplifying the process of going from cells to tissues using statistical mechanics

1 CONSTRUCTION OF A NETWORK MODEL OF CARDIAC VENTRICULAR TISSUES

Consider the digital reconstruction of a cardiac ventricular tissue segment to simulate the conduction of electrical wave, Figure S1. Here, 3D confocal image of region of rat cardiac ventricular tissue with myocardial infarct was imaged using confocal microscopy at 0.5μ m resolution. The image was then tessellated into a mesh of conforming voxel units. Following this, the discrete volumes and representative nodal locations of surviving myocardium within each unit were determined. Since electrical conduction is dominated by myocyte fiber tracts, this observation is used to generate the interconnection network. Multiple discrete myocyte fiber tracts through a voxel unit are treated as independent network components and are used to generate a discrete network of myocytes. The nodes of the network are assigned local properties like nodal volumes, inter-volume connection edge areas, distances between adjacent nodes, etc.

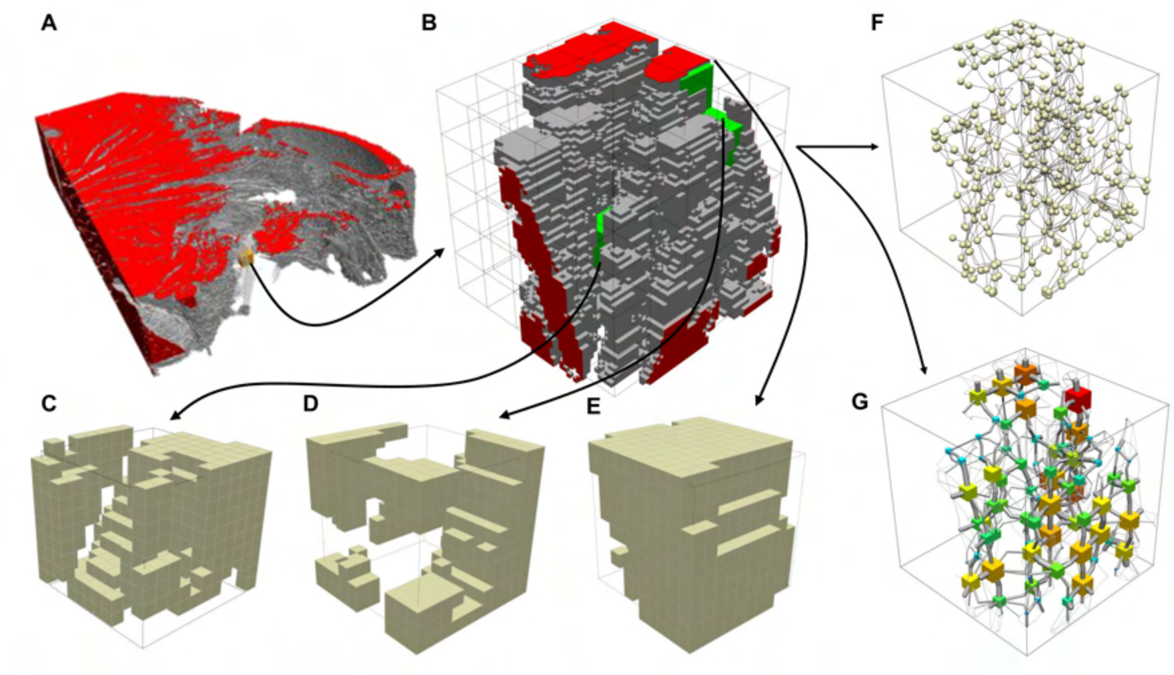

Figure S1. Discretisation of tissue image to define computational mesh and cellular connectivity network. A. The location of the example block in the full 3D rat myocardial infarct image. B. The isolated example block comprised of 51×51×51 voxels (0.5μ m resolution). C-E. The example block is subdivided into 5×5×5 sub-blocks, each of size 11×11×11 voxels. The sub-blocks overlap neighbouring sub-blocks by one voxel. The surfaces where the isolated example block connects with the larger image are highlighted in green. F. Tissue interconnection network showing nodes (myocytes) and edges (physical connections). D. Augmented network showing volumes (property) assigned to each node and edges scaled to reflect relative cross-section.

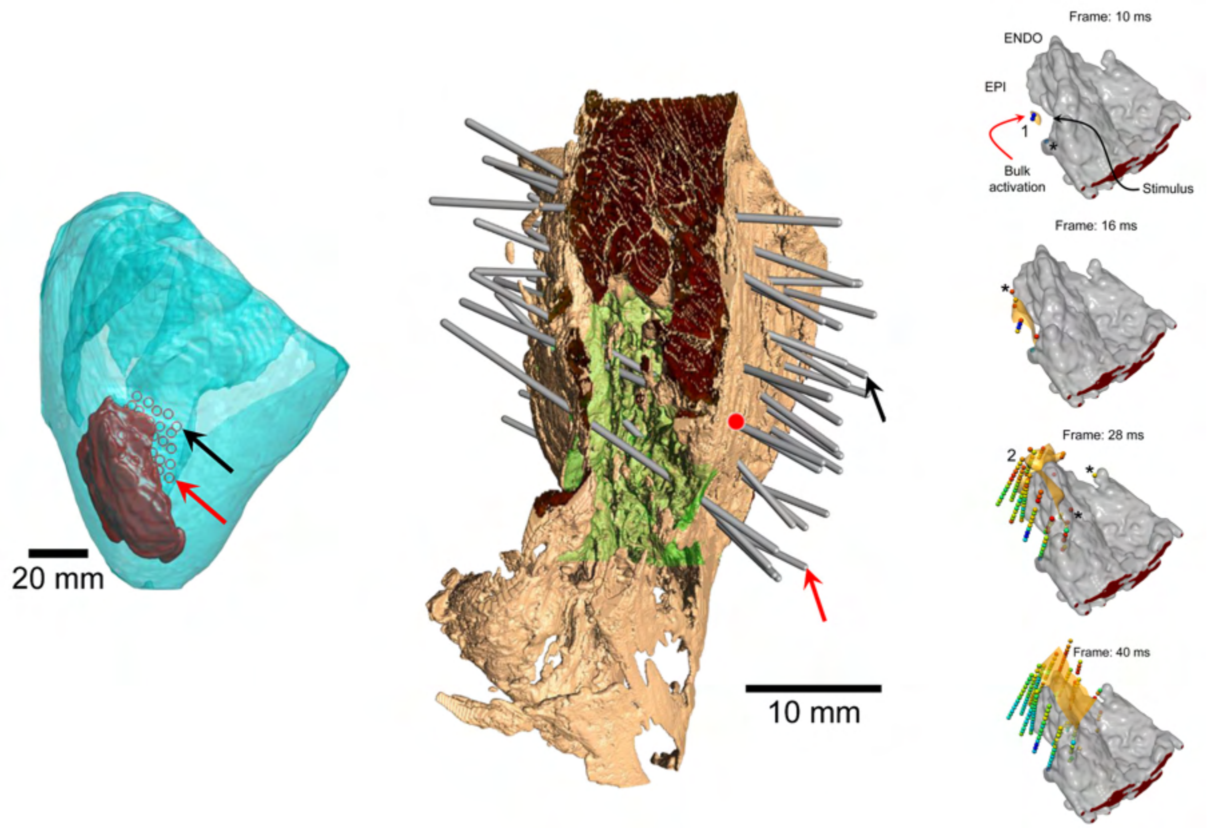

Figure S2. Left: Plunge electrode reconstruction of electrical wave propagation in infarct tissue(red) and left ventricular tissue (blue). Middle: Three-dimensional reconstruction of myocardium (dark red), plunge needles (silver), and infarct boundary (green) segmented from serial block surface images of heart. Location electrodes relative to the whole heart are shown by the black and red arrows. Right: Three-dimensional propagation of electrical activation following subepicardial stimulation near the border zone of the heart. Activation times at electrodes and interpolated -$10mV$ potential isosurfaces (gold) are shown at 10, 16, 28, and 40 ms after stimulation 0.9 mm outside the infarct boundary.

Typically, characterisation of tissue level processes involves simultaneous measurement of events (signals, chemical/ionic potentials etc.) across the tissue. For instance, through plunge electrodes that measure ionic fluctuations when excitory waves propagate (Trew et al., 2019). Figure S2, shows one such process of characterising the tissue electrical conduction dynamics. Data related to the dynamics, such as correlation of conduction velocities, activation times etc. are mapped on to the network (see Figure S1G).

2 BONDGRAPH MODEL OF CARDIAC ACTION POTENTIAL COMPOSED USING SUBSCALE MODELS

Pan et al. (2018) present a detailed bondgraph model of cardiac action potential, including the demonstration of composing this model from bondgraph models that characterise the subscale processes. Here we highlight the key elements of the model in figure S3 and its ability to reproduce action potentials in figure S4. The interested reader is referred to (Pan et al., 2018) for details.

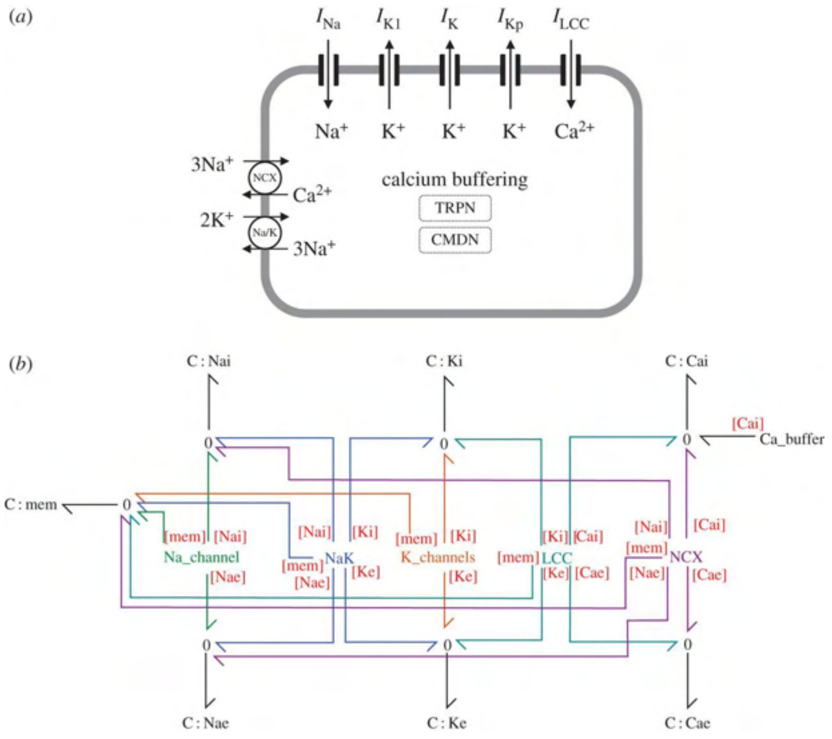

Figure S3. Action potential model. (a) Cell schematic, (b) overall bond graph structure. The bond graph modules Na_channel, NaK, K_channels, LCC, NCX and Ca_buffer contain more detailed aspects of the bond graph structure Definitions: I_{Na}, sodium current; I_{K1}, time-independent potassium current; I_K, time-dependent potassium current; I_{Kp}, plateau potassium current; I_{LCC}, L-type Ca_{2+} current; NCX, $Na^+ - Ca^{2+}$ exchanger; Na/K, Na^+/K^+ATPase; TRPN, troponin; CMDN, calmodulin.
Reproduced Figure 1 with permission from Pan et al. (2018)

3 FEASIBILITY DEMONSTRATION

3.1 Substrate dependent excitation propagation model

We use the model developed by Christensen et al. (2015) for electrical fibrillation as a datasource to construct and analyse ERGMs for various structural configurations. The model characterises the formation of rentrant circuits due to lateral uncoupling of myocardial tissue. The level of structural heterogeneity is controlled by ν, a model parameter that determines the fraction of cells with lateral cell-to-cell coupling. Activation dynamics is modelled using a simple finite state automaton that switches its states based on current state, refractory period and excitatory state of the neighbouring cell, Figure S5.

At low levels of structural heterogeneity wave propagation remains planar. However, beyond a threshold value, spontaneous localised disruption of wave propagation and degeneration to fibrillation is observed, Figure S6. Their model allows us to analytically calculate risk of fibrillation as a function of structural heterogeneity ν, and therefore well suited for analysing structural complexity.

For the purpose demonstration, We generated 2D tissue configurations for $\nu = 0.1, 0.2, 0.3, 0.4, 0.5, 0.6$ $, 0.7, 0.8, 0.9$ with $L = 200$. The models were simulated with $\tau = 50 \pm 5$, probability that dysfunctional cells may fire on excitation $\epsilon = 0.05$, and the fraction of dysfunctional cells $\delta = 0.05$ to determine the excitory wave propagation.

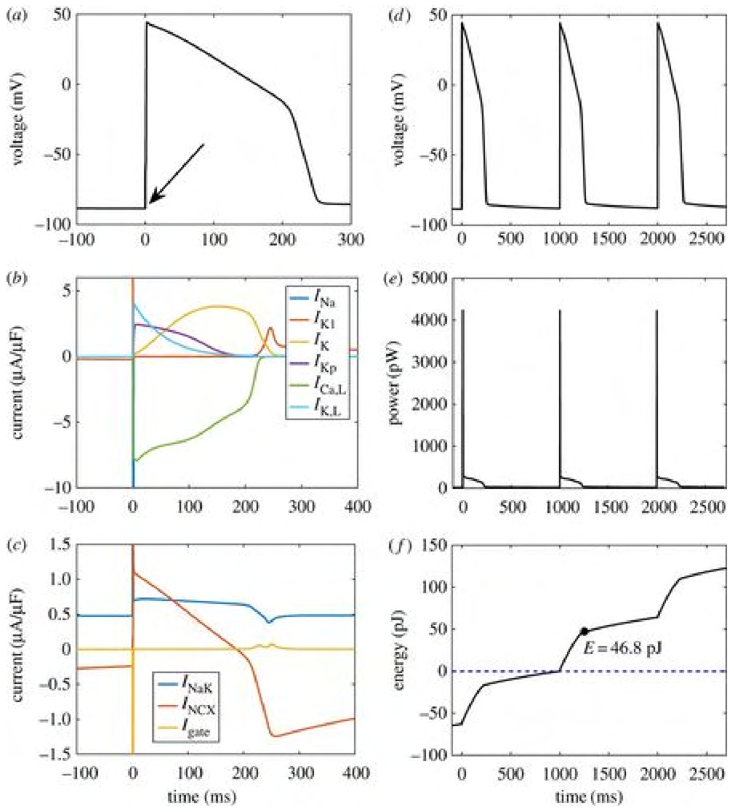

Figure S4. A simulation of the cardiac action potential using a bond graph model. (a) Membrane voltage, following stimulation with a conservative stimulus current (arrow); (b) ion channel currents; (c) transporter and gating currents; (d) membrane voltage over three cycles, for comparison with (e) and (f); (e) power consumption; (f) energy dissipated, with the variable E representing the energy consumption over the duration of the action potential. The model was run initially for 300ms to allow the membrane potential and channel gates to stabilize. The intracellular ion concentrations were dynamic variables with initial concentrations $[Na_i^+] = 10mM$, $[K_i^+] = 145mM$ and $[Ca_i^+] = 0.12M$. Constant concentrations were $[Na_e^+] = 140mM$, $[K_e^+] = 5.4mM$, $[Ca_e^+] = 1.8mM$, $[MgATP] = 6.95mM$, $[MgADP] = 0.035mM$, $[Pi] = 0.3971mM$ and $pH = 7.095$. $T = 310K$.
Reproduced Figure 7 with permission from Pan et al. (2018)

3.2 Network model

There are 200^2 cells in the 2D tissue and algorithms to fit ERGM's struggle with such large networks. Following experimental approaches like the plunge electrode measurements discussed above, a uniformly spaced sparse grid of 10×10 nodes, was place on the 2D tissue and the local state transition kinetics (averaged over a 3×3 element neighbourhood around the element) and structural properties like number of lateral connections was sampled. The sampled locations were defined as the network nodes, the average local lateral connectivity value was used as the node weight, and directed edges were created using the state transitions kinetics. Specifically, we estimated the granger causality using partial correlations (Runge et al., 2019) with a lag of 4 times the average refractory period $\tau = 50$ units. Similar methods have been used to create brain networks/connectomes (Sanz-Leon et al., 2015).

3.3 GERGM

There are several variants of ERGMs. Here we used Generalized ERGM (GERGM). GERGMs are analogous to the standard logistic and linear regression frameworks, but with the network structure as the

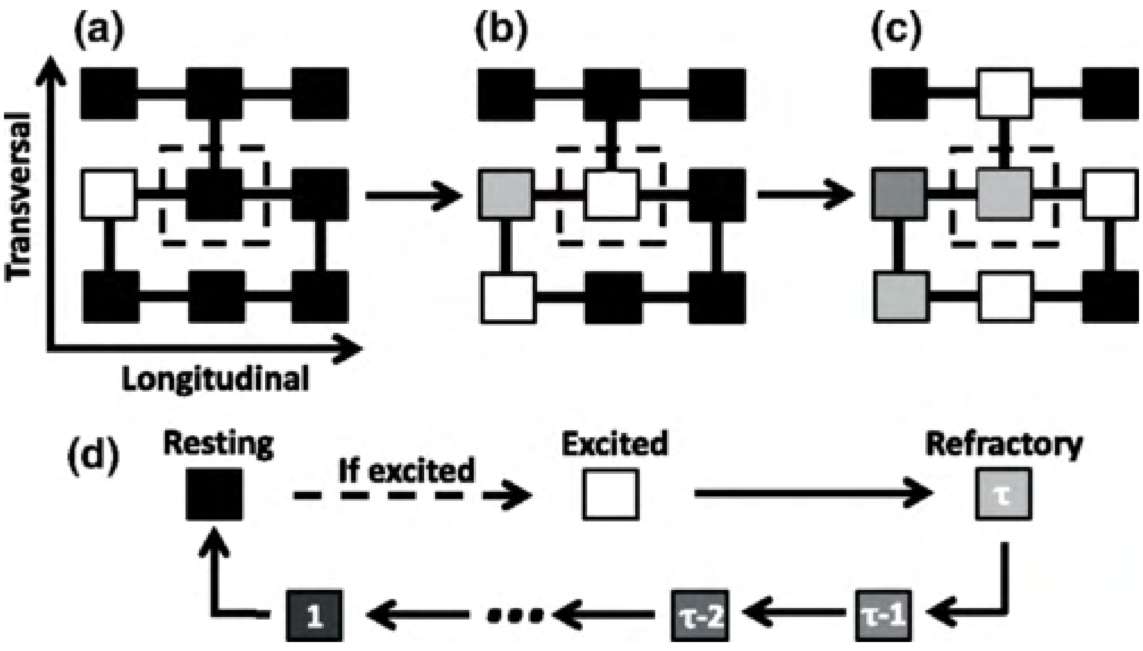

Figure S5. A simple lattice based model of electrical conduction. Coupling is represented by a link between two cells. Cells are always coupled longitudinally and with frequency ν transversely. (a),(b) A resting cell (black) will become excited (white) in the next time step if at least one of its neighbouring coupled cells are excited. (b),(c) Once a cell is excited it will enter a refractory state (grey) for a duration of time τ. (d) The time course of a cell once it has been excited by an excited neighbouring coupled cell. Reproduced Figure 1 of (Christensen et al., 2015).

dependent variable (Desmarais and Cranmer, 2012). GERGMs fit models by generating networks with similar structural, node and edge attribute weight characteristics. Here, three network motifs relevant to arrhythmic risk were specified as variables: (i) nodes with two incoming network links (*in2stars*), (ii) nodes with two outgoing network links (*out2stars*), and (iii) nodes forming local cyclic networks with two other nodes (*ctriads*). Nodes functioning as hubs with either *Sender* or *Receiver* effects were additional constraints on the GERGM models. GERGM models using these three network motif variables were fit to the original networks $\mathcal{G}(\nu)$, constructed in section 3.2, to find three weight parameters (θ_1, θ_2 and θ_3) for the observed motif counts (x_1, x_2, x_3). These θ_i are the parameter values for the distribution, from which the algorithm generates candidate networks, that maximises the likelihood of observing/generating a network with the specified node and edge weights.

A Markov Chain Monte Carlo method chooses θ_i to maximize the likelihood of generating networks with edge weights (”network covariate”) similar to $\mathcal{G}(\nu)$ (the cross-correlation of average cell state time series between connected nodes), and nodes with weights (”con”) similar to $\mathcal{G}(\nu)$ (the average local-lateral connectivity). The ERGM expression of this objective is given below:

net $\sim$ *edges + netcov(”network covariate”) + sender(”con”) + receiver(”con”) + out2stars(”con”) + in2stars(”con”) + ctriads*

Existing software tools were used as a black box to solve these problems (Denny, 2016).

3.4 Simulation code

The code used to simulate 2D tissues, generate the networks and fit GERGM models are available at https://github.com/Jagirhussan/GERGMINCARDIACELECTROPHYSIOLOGY

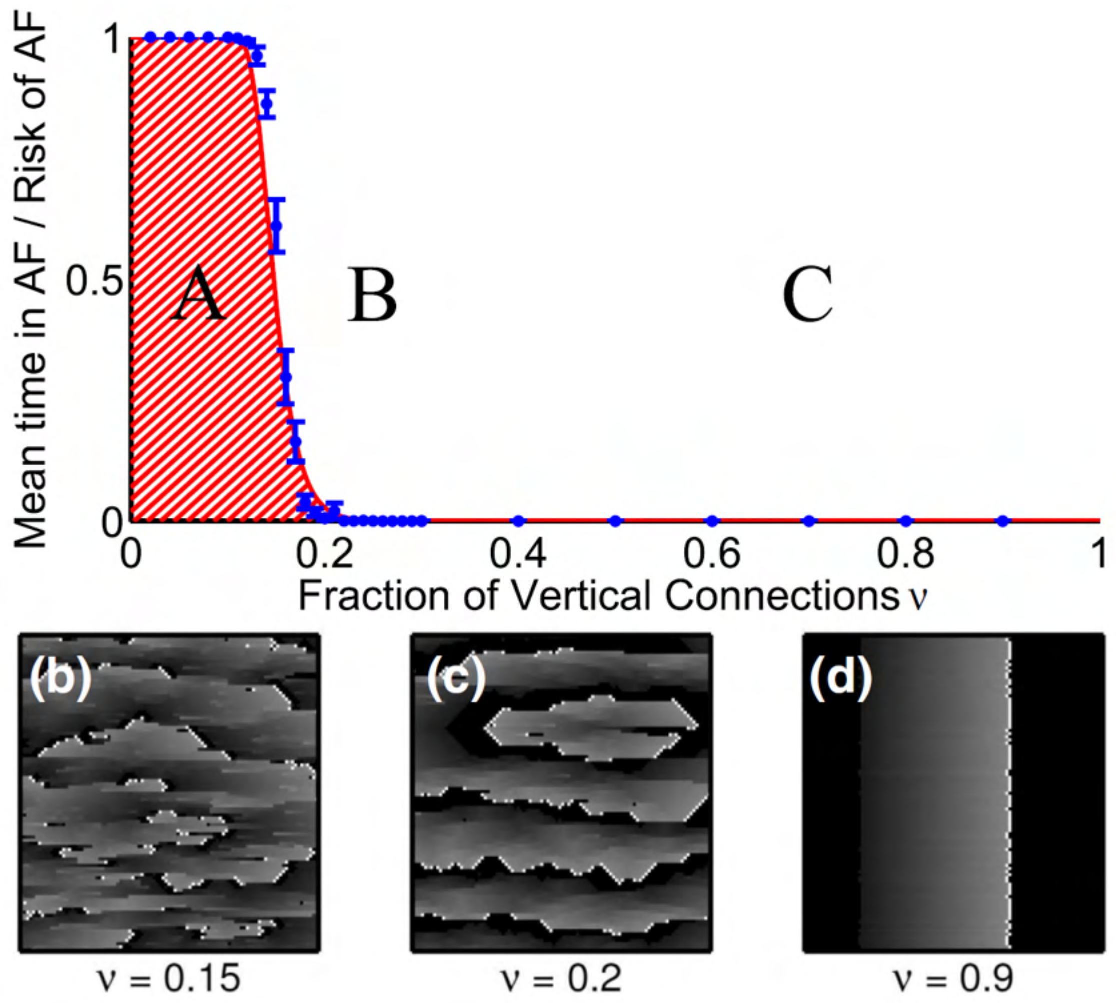

Figure S6. As ν decreases from 1, the excitation propagation transition from planar wave fronts (C) to a few self-terminating reentrant circuits (B). As ν decrease below 0.14 the system develops multiple self-sustaining reentrant circuits (A). The red line is the analytically calculated probability having at least one fibrillation-inducing structure. The solid blue circles represent the average duration that a system displays nonplanar wave fronts, averaged over 50 realisations of duration 106 timesteps and error bars represent one standard error of the mean. (b)–(d) Realisations of systems with $\nu = 0.9$ behaviour,$\nu = 0.2$—single self-terminating rotor, and $\nu = 0.15$—multiple self-terminating rotors.
Reproduced Figure 3 of (Christensen et al., 2015).

4 SIMULATION RESULTS

High resolution images of the excitation state of the 2D tissue for at $T = 1000$ the various ν values in Figure 2 of the main text. Here, τ is the refractory period, ϵ is the probability that the cell may not excite, and δ is the fraction of dysfunctional cells in the tissue.

The GERGM coefficients, structural parameters and potentials are listed below:

ν	in2stars	out2stars	ctriads	θ_1	θ_2	θ_3	ERGM Potential
0.1	1.08E+04	8.21E+03	1.71E+03	3.53E-01	7.47E-01	-1.65E+00	8.14E+03
0.2	6.36E+04	6.31E+04	1.79E+04	-1.08E-02	-1.95E-03	7.02E-02	4.51E+02
0.3	7.21E+04	7.20E+04	2.14E+04	-1.25E-02	-9.00E-03	8.29E-02	2.25E+02
0.4	7.75E+04	7.78E+04	2.35E+04	-1.20E-02	-1.22E-03	8.80E-02	1.04E+03
0.5	8.03E+04	8.02E+04	2.47E+04	-1.13E-02	-1.40E-02	9.23E-02	2.49E+02
0.6	6.53E+04	6.65E+04	2.00E+04	-1.49E-03	-1.69E-03	1.17E-01	2.13E+03
0.7	7.43E+04	7.27E+05	2.19E+04	2.00E-03	1.10E-02	5.90E-02	3.56E+03
0.8	7.95E+04	7.88E+04	2.36E+04	3.77E-03	1.89E-02	2.26E-02	2.33E+03
0.9	6.82E+04	7.02E+04	2.39E+04	-2.03E-03	-8.67E-03	1.85E-01	3.69E+03

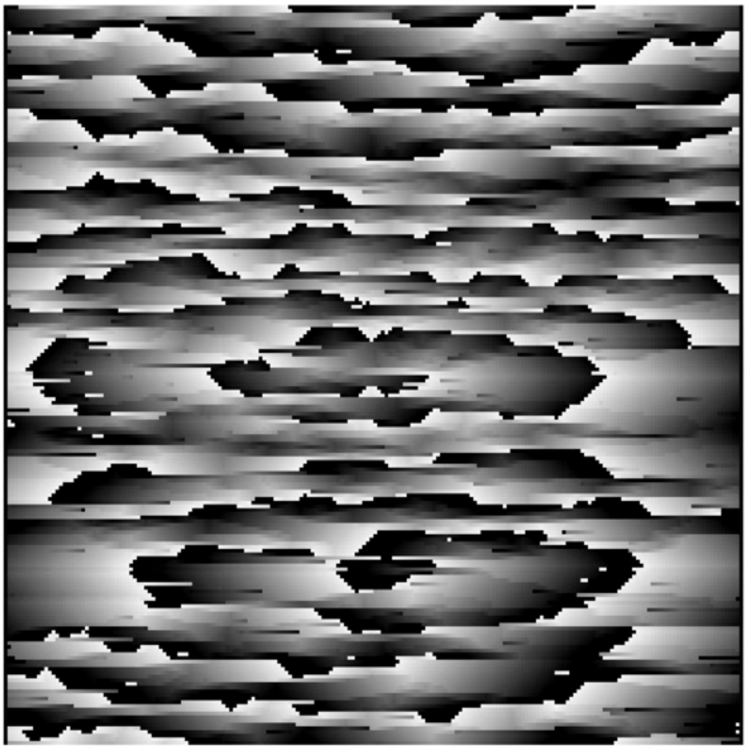

Figure S7. Excitation propagation wave fronts at $T = 1000$, $\nu = 0.1$, $\tau = 50 \pm 5$, $\epsilon = 0.05$, and $\delta = 0.05$

REFERENCES

Christensen, K., Manani, K. A., and Peters, N. S. (2015). Simple model for identifying critical regions in atrial fibrillation. *Phys. Rev. Lett.* 114, 028104. doi:10.1103/PhysRevLett.114.028104

[Dataset] Denny, M. J. (2016). Generalized Exponential Random Graph Models. `https://github.com/matthewjdenny/GERGM.git`. [Online; accessed 21-October-2021]

Desmarais, B. A. and Cranmer, S. J. (2012). Statistical inference for valued-edge networks: the generalized exponential random graph model. *PLoS One* 7, e30136

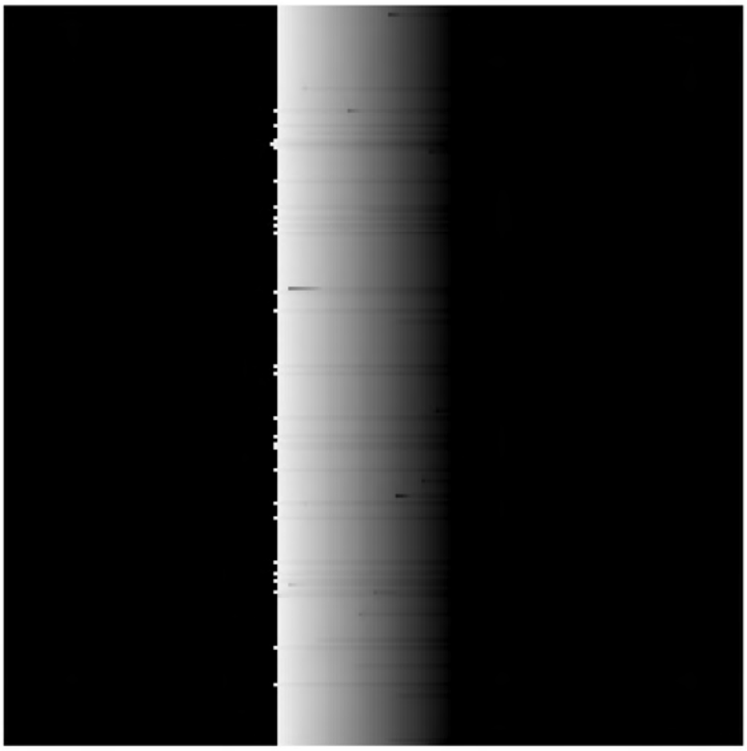

Figure S8. Excitation propagation wave fronts at $T = 1000$, $\nu = 0.2$, $\tau = 50 \pm 5$, $\epsilon = 0.05$, and $\delta = 0.05$

Pan, M., Gawthrop, P. J., Tran, K., Cursons, J., and Crampin, E. J. (2018). Bond graph modelling of the cardiac action potential: implications for drift and non-unique steady states. *Proc Math Phys Eng Sci* 474, 20180106

Runge, J., Nowack, P., Kretschmer, M., Flaxman, S., and Sejdinovic, D. (2019). Detecting and quantifying causal associations in large nonlinear time series datasets. *Sci Adv* 5, eaau4996

Sanz-Leon, P., Knock, S. A., Spiegler, A., and Jirsa, V. K. (2015). Mathematical framework for large-scale brain network modeling in The Virtual Brain. *Neuroimage* 111, 385–430

Trew, M. L., Engelman, Z. J., Caldwell, B. J., Lever, N. A., LeGrice, I. J., and Smaill, B. H. (2019). Cardiac intramural electrical mapping reveals focal delays but no conduction velocity slowing in the peri-infarct region. *American journal of physiology. Heart and circulatory physiology* 317, H743–H753

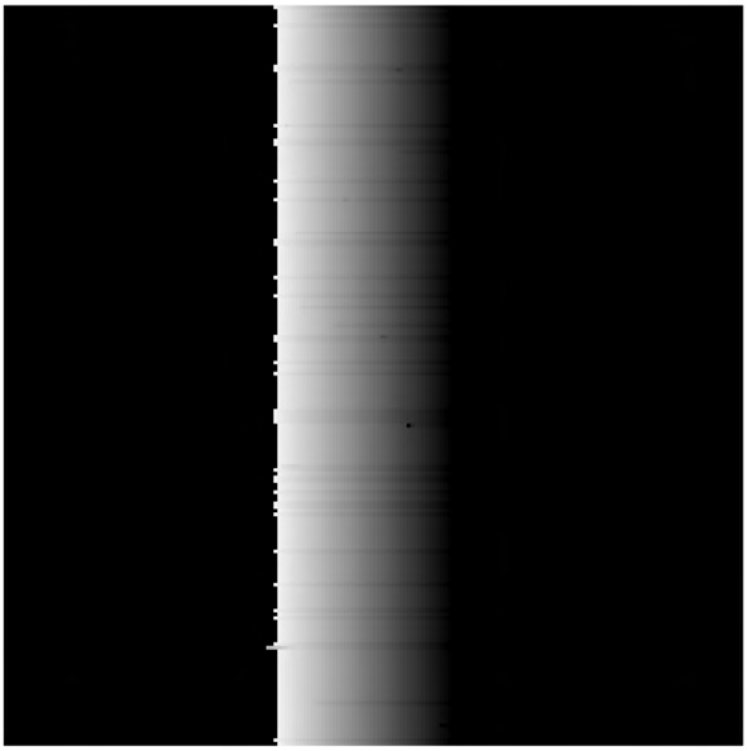

Figure S9. Excitation propagation wave fronts at $T = 1000$, $\nu = 0.3$, $\tau = 50 \pm 5$, $\epsilon = 0.05$, and $\delta = 0.05$

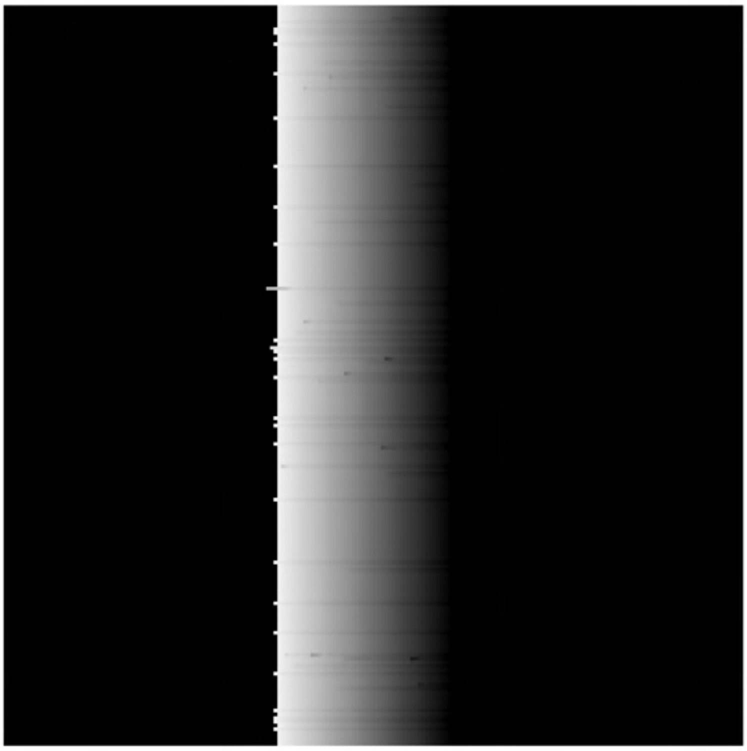

Figure S10. Excitation propagation wave fronts at $T = 1000$, $\nu = 0.4$, $\tau = 50 \pm 5$, $\epsilon = 0.05$, and $\delta = 0.05$

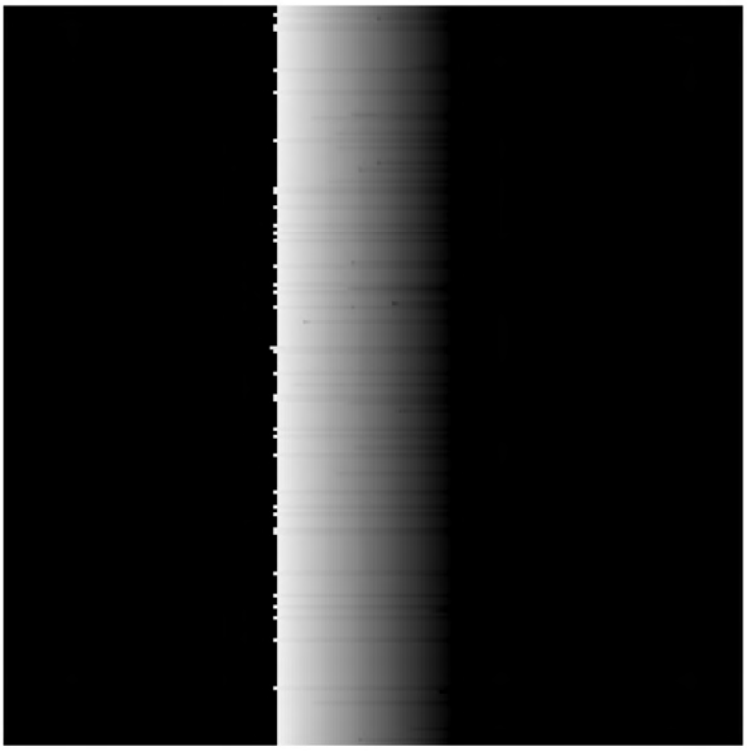

Figure S11. Excitation propagation wave fronts at $T = 1000$, $\nu = 0.5$, $\tau = 50 \pm 5$, $\epsilon = 0.05$, and $\delta = 0.05$

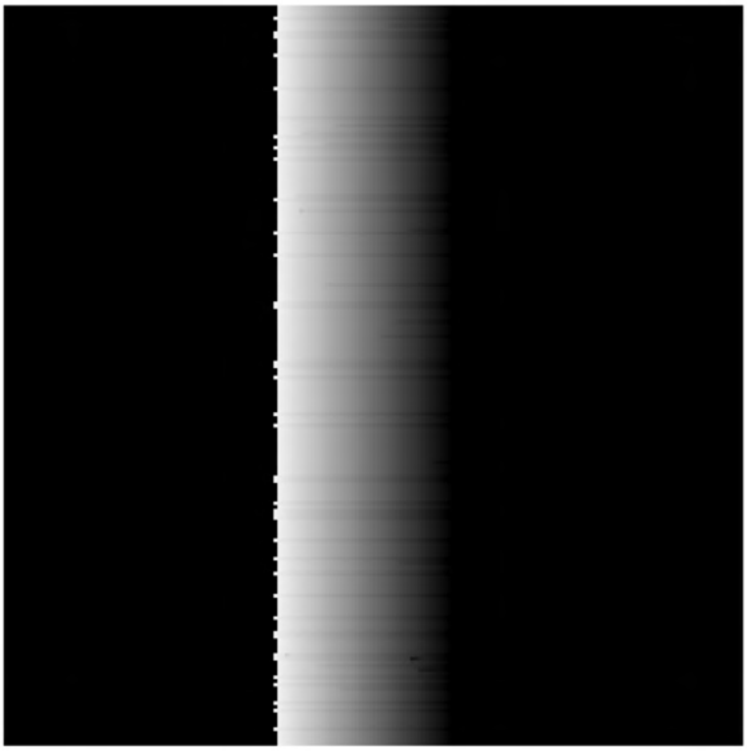

Figure S12. Excitation propagation wave fronts at $T = 1000$, $\nu = 0.6$, $\tau = 50 \pm 5$, $\epsilon = 0.05$, and $\delta = 0.05$

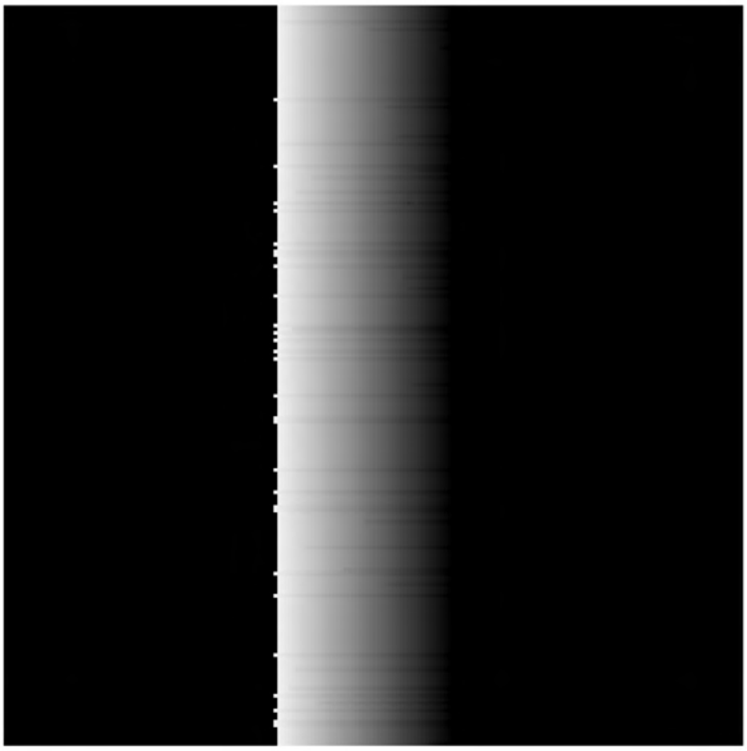

Figure S13. Excitation propagation wave fronts at $T = 1000$, $\nu = 0.7$, $\tau = 50 \pm 5$, $\epsilon = 0.05$, and $\delta = 0.05$

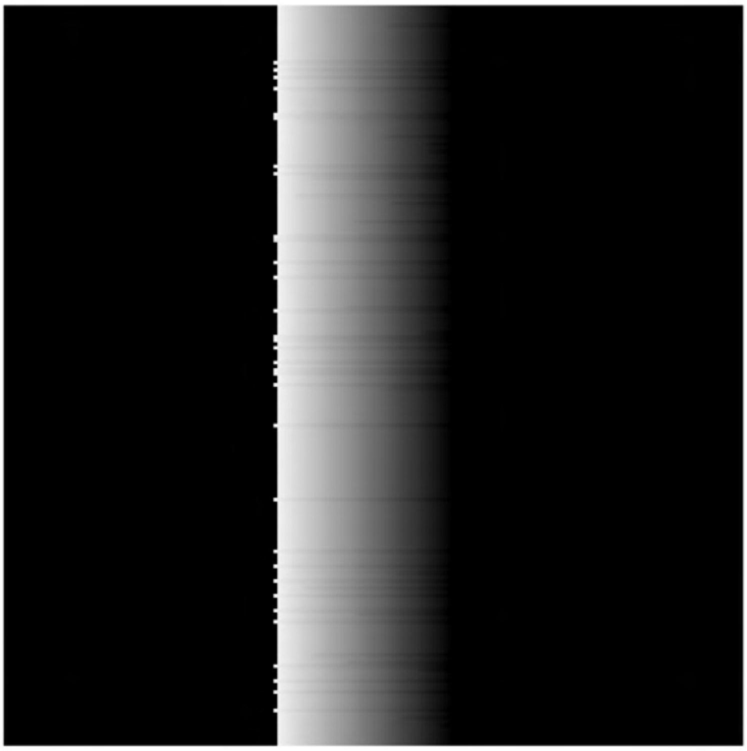

Figure S14. Excitation propagation wave fronts at $T = 1000$, $\nu = 0.8$, $\tau = 50 \pm 5$, $\epsilon = 0.05$, and $\delta = 0.05$

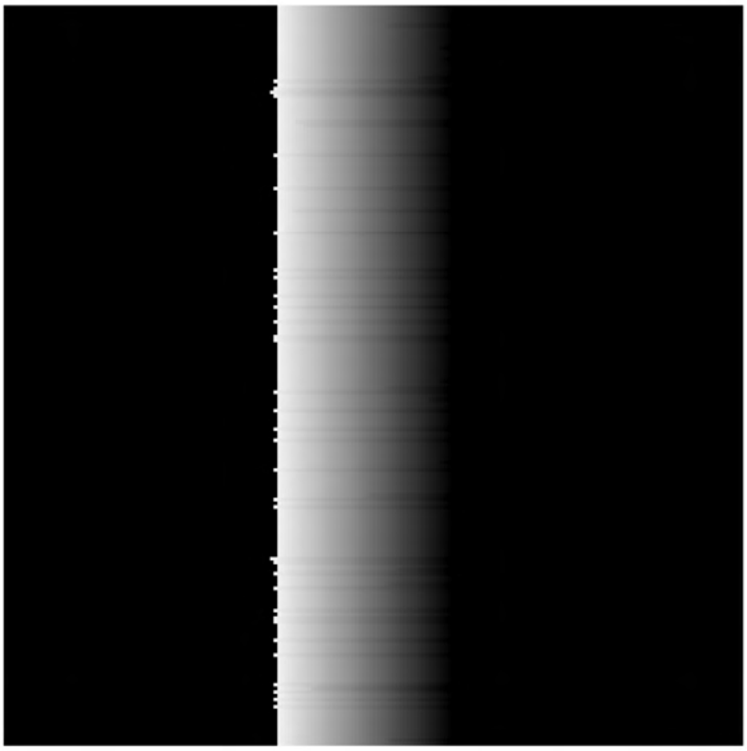

Figure S15. Excitation propagation wave fronts at $T = 1000$, $\nu = 0.9$, $\tau = 50 \pm 5$, $\epsilon = 0.05$, and $\delta = 0.05$
